# Medical terminology in online patient–patient communication: evidence of high health literacy?

**DOI:** 10.1111/hex.12395

**Published:** 2015-08-19

**Authors:** Antoinette M. Fage‐Butler, Matilde Nisbeth Jensen

**Affiliations:** ^1^ Department of Business CommunicationAarhus UniversityAarhusDenmark

**Keywords:** chronic disease, e‐patient, health literacy, medical terminology, online forum, patient–patient communication

## Abstract

**Background:**

Health communication research and guidelines often recommend that medical terminology be avoided when communicating with patients due to their limited understanding of medical terms. However, growing numbers of e‐patients use the Internet to equip themselves with specialized biomedical knowledge that is couched in medical terms, which they then share on participatory media, such as online patient forums.

**Objective:**

Given possible discrepancies between preconceptions about the kind of language that patients can understand and the terms they may actually know and use, the purpose of this paper was to investigate medical terminology used by patients in online patient forums.

**Design:**

Using data from online patient–patient communication where patients communicate with each other without expert moderation or intervention, we coded two data samples from two online patient forums dedicated to thyroid issues.

**Results:**

Previous definitions of medical terms (dichotomized into technical and semi‐technical) proved too rudimentary to encapsulate the types of medical terms the patients used. Therefore, using an inductive approach, we developed an analytical framework consisting of five categories of medical terms: dictionary‐defined medical terms, co‐text‐defined medical terms, medical initialisms, medication brand names and colloquial technical terms. The patients in our data set used many medical terms from all of these categories.

**Discussion and conclusions:**

Our findings suggest the value of a situated, condition‐specific approach to health literacy that recognizes the vertical kind of knowledge that patients with chronic diseases may have. We make cautious recommendations for clinical practice, arguing for an adaptive approach to medical terminology use with patients.

## Introduction

Inappropriate use of medical terms in health‐care professional–patient communication has been associated with problems in relation to patient empowerment, patient autonomy, patients’ emotional ease, satisfaction and compliance.^1^, ^2^, ^3^, ^4^ Of particular concern is professionals’ use of expert *jargon*,^5^, ^6^ as patients may not understand it.^1^, ^7^, ^8^, ^9^ Patients may employ different lexical items than health‐care professionals for the same concept,^3^ also referred to as *patientese*,^5^ or alternatively associate different quantities as well as kinds of information with specialist terms.^8^, ^10^ In keeping with such concerns, health‐care professionals are often advised to avoid medical terminology when communicating with patients^1^, ^2^, ^11^; instead, they should translate medical jargon into lay‐friendly lexis.^6^

The on‐going relevance of such general recommendations requires investigation in the light of the fact that growing numbers of patients are accessing health resources on the Internet,^12^ gaining familiarity with medical terms in the process.^13^, ^14^ Indeed, both a recent collaboration^15^ and Armstrong *et al*.'s study^16^ indicated that patients share technical and biomedical knowledge on diagnosis, managing illness and treatment online – knowledge that would need to be mediated via terminology. Over a decade ago, Bowker & Herrera,^17^ as part of their linguistic study, found the use of medical terms in an online patient forum, and this is likely to be more pronounced now. Also, many patients actually appreciate that their health‐care professionals use medical terminology,^18^ describing ‘appropriate and consistent medical terms’ as contributing to positive health‐care experiences.^19^

Unlike previous studies, such as Antelmi^20^ and Jucks & Bromme^10^ that examined the use of terms in *doctor–patient* online communication, this paper investigates patients’ use of medical terms in what we have previously termed *patient–patient* online communication,^15^ where communication is not mediated by a health‐care professional, and where patients communicate with each other without expert moderation or intervention. By focusing on naturally occurring patient–patient communication, not only are we addressing a significant empirical research gap, but we are also able to avoid a potential pitfall as health‐care professionals might introduce medical terms that patients would not use as part of their normal vocabulary. This is a relevant concern as patients are more likely to use medical terms when communicating online with doctors than with patients.^20^ Besides its empirical aspirations, this paper aimed to promote theoretical understandings of patients’ evolving health literacy, given that it may be positively affected by Internet use.^21^ Understanding developments in patients’ health literacy is important, as high health literacy is associated with greater patient confidence^22^ and greater patient participation in health.^23^ The fact that health literacy is promoted through supportive social networks – which Edwards *et al*.^24^ describe as ‘distributed health literacy’ – is very relevant for the online forum setting, where patients learn from each other.

The paper begins with a review of theoretical perspectives on health literacy in relation to patients’ use of medical terms before discussing the *e‐patient*, a categorization of the patient that is relevant for this paper given its use of online data. The research design is then described: our data set drawn from two online patient forums, and our analytical framework, which supports the identification and characterization of medical terminology. After this, our findings are presented and discussed. We conclude with suggestions for future research and highlight the practical implications of our findings for communication in the clinical setting.

## Patients and medical terminology

### Health literacy and terminology

Health literacy is a multifaceted and evolving construct, subsuming health literacy types such as functional, interactive and critical.^25^ Theories of functional health literacy provide the framework for the present study. Functional health literacy relates to the ability to understand and use health information^26^; as such, it incorporates patients’ understanding of medical terms.^27^ Such understanding presupposes some conceptual knowledge of health‐related topics, with terminology being the means by which this knowledge is communicated, as terms allow people ‘not only to communicate and interact, but also to transfer their knowledge with a didactic purpose to train new experts, or simply spread special knowledge as information to the general public willing to learn about a subject’.^28^ Here, Cabré underlines the potential for terminology to increase participation and engagement; Ferguson^29^ similarly associates knowledge of medical terminology with empowerment.

Reflecting the importance of medical terms to health literacy, the most popular tests of health literacy assess patients’ ability to manage or understand terms. REALM, for example, focuses on whether patients can pronounce a term correctly,^30^ SAHLSA requires patients to view a stimulus term and choose which of two other words is most similar in meaning,^31^ and the reading subtest of TOFHLA asks patients to choose the correct term from a range of terms.^32^ Elder *et al*.^33^ identify weaknesses in these tests, identifying REALM as measuring familiarity as opposed to understanding; they also suggest that TOFHLA measures literacy factors that affect health outcomes rather than health literacy *per se*. The link between poor understanding of medical terminology and low functional health literacy is also evident in studies that identify patients with poor health literacy as struggling to understand medical terms.^34^, ^35^ A lack of understanding of medical terms can explain how even highly literate people can lack functional health literacy.^36^

Most often in the literature, health literacy is described as *low*; deficit models of patients are invoked, and empirical investigations into high health literacy are rare. This paper is open to the possibility that some patients may have high health literacy, which may be exemplified by their use of medical terminology.

### Health literacy and e‐patients

A working assumption of patients’ lack of understanding of terminology, specifically, and their laity, more generally, may have benefits, as assuming that patients comprehend medical terms, when this is not the case, can jeopardize communication and thus health outcomes,^7^ as well as be ethically dubious. However, certain groups of patients are increasingly equipping themselves with biomedical information by using the Internet, as the Internet makes biomedical information available that once was inaccessible to the public.^14^ This has led to concerns about the digital divide and technology's role in exacerbating health inequalities.^37^ However, with increasing numbers of patients using the Internet for health searches, there is the sense of a paradigmatic shift: in line with the trend towards e‐health,^38^ the *e‐patient* is considered to be replacing the *patient* of the biomedical model.^29^, ^39^ Given the role they play in managing their own conditions, it is argued that e‐patients should be recognized as health‐care professionals’ medical collaborators; indeed, e‐patients’ biomedical knowledge of their own condition can in some cases surpass that of health‐care professionals.^29^ It should be underlined that the kind of knowledge that patients have is thus not restricted to their ‘experiential knowledge’,^40^ but can actually include biomedical knowledge, such as knowledge of medical terms. For the purpose of this paper, we define e‐patients as availing of information technologies to equip themselves with relevant condition‐related knowledge and support; thus, all the patients who are involved in producing the threads in the data set of this paper are e‐patients.

## Methods

### Analytical framework: medical terms and how to identify them

For early terminologists like Wüster,^41^ terms were easily distinguishable from (ordinary) words because terms related to concepts within a specialized field, whereas words did not. However, the word/term dichotomy envisaged by Wüster has been dismantled by middle ground concepts such as semi‐technical terms, the distinction between medical technical terms and medical everyday terms,^42^ the idea that medical language should be seen as lying along a continuum^43^ and Nation's^44^ notion of technicalness as a spectrum which has to do with ‘how restricted a word is to a particular area’. Furthermore, pragmatic context is increasingly recognized as playing an important role in identifying whether a word is a term.^45^ Interestingly, the distinction between words and terms is completely eroded by Cabré Castelví,^45^ who suggests that any lexical unit can potentially be a terminological unit.

The fuzziness – even dissolution – of the term/word border left us with the practical methodological challenge of operationalizing the notion of a medical term for our empirical study. Our initial review of the literature suggested that medical terminology consisted of two types: (i) technical and (ii) semi‐technical. Technical terms, which are often Latin or Greek in origin, or relate to medication names, can be identified using medical reference books or dictionaries,^7^, ^10^, ^46^, ^47^ whilst semi‐technical terms may be identified using definitions and examples.^8^, ^9^, ^47^ Drawing on this distinction, we used technical and semi‐technical terms as categories for our preliminary analytical framework.

### Data

To investigate patients’ use of terminology, we chose online patient forums where patients communicate online with other patients about a particular health condition. In such forums, a patient commences a thread by posting a comment, question or story, to which other members then reply.^48^

We chose a thyroid forum for our study for a number of reasons. First, health resources on the Internet are especially relevant for patients with chronic diseases for whom relevant information on self‐management and coping strategies are particularly valuable.^49^ Second, thyroid issues require patients to be proactive, in that self‐care, such as daily medication, must be administered; the discussion of thyroid issues would also include thyroid cancer, where patients would be highly motivated to understand their condition and its treatment.^50^ Third, clinical presentations of thyroid hormone deficiency are diverse, complicated and often overlooked,^51^ with the onset of hypothyroidism being ‘so insidious that even classic symptomatology may go unnoticed or undiagnosed’.^52^ Given the sharing of information and experiences possible on online forums, thyroid patients may obtain particular benefits from participating on patient forums, which may be reflected in their use of terminology.

We chose to investigate publicly available forums, where membership is not required to view forum threads. Unmoderated online patient–patient forums provide access to live and authentic interactions between patients, circumventing some of the problems involved in using data derived from artificial, data‐generating situations such as experiments or interviews. Such forums also make it possible to investigate instances of health communication in what Jones^53^ refers to as ‘in the wild’. Moreover, as suggested by Godbold,^54^ in such forums, patients may be less self‐censored than in institutional forums. As we used public domain data, the study did not require ethics approval.

### Analytical procedure

Our analysis followed the process below:

1. We pilot‐tested the above‐mentioned framework of medical terms being either technical or semi‐technical using one sample (Sample 1),
2. We fine‐tuned the framework following the pilot‐test, and
3. We coded Sample 1 again, augmenting the study with a second, larger sample (Sample 2).

Sample 1 consisted of a thread from the general discussion area of an online thyroid forum. This thread was chosen because: (i) the general area of the forum was likely to attract both newcomers and regular, long‐term users, (ii) it was initiated by a patient recently diagnosed with thyroid cancer due to have a thyroidectomy, which made it likely that medical terms would be present, and (iii) it had 45 replies with nine different contributors, who had various levels of experience with the forum as measured by the number of posts each contributor made, ranging from 16 to 17 253 posts. The large range was another important reason for choosing this thread, as we wanted to ensure the inclusion of both seasoned posters and contributors who were new to posting. This sample consisted of 2387 words. All posts were coded by the two authors independently, and all identified terms were subsequently checked to ensure agreement.

We quickly found that the categories of technical and semi‐technical terms were lacking; firstly, *semi‐technical* was not very useful for our data and research question, as its scope – it includes words which are ‘minimally related to the field of anatomy’, such as ‘part’ and ‘pairs’,^55^ as well as words like ‘eye’ and ‘neck’^47^ – did not reflect the *expert medical* terms that we were interested in investigating. Secondly, *technical* proved to be too open as a coding category. Thus, working with Sample 1, we identified on an inductive and collaborative basis the need for further refinement in the form of subcategorization and for an increase in the number of categories.

As for subcategorization, we found it useful to subdivide medical terms into *dictionary‐defined medical terms* and *co‐text‐defined medical terms*. The first category of dictionary‐defined terms includes Latin‐ and Greek‐derived terms as well as those that are not, leading to a category that captured terms like *Papillary carcinoma thyroid cancer* and *collar bone*. To determine whether these were medical terms, we used Stedman's Medical Dictionary (online) and MedlinePlus medical dictionary (online) (see Table 1). The second category of co‐text‐defined medical terms was necessary to capture terms like *uptake* and *replacement*, which were not found in the medical dictionaries, and which have a non‐specialized meaning, but when used in a medical context, carry specialized meaning. Co‐text‐defined terms are thus terms which overlap expert and general language.

**Table 1:** Categories of medical terms found in our data and methods used

	Term type	Examples	Method
1.	Dictionary‐defined medical terms	Papillary carcinoma thyroid cancer Collar bone	Medical dictionaries http://www.drugs.com/medical_dictionary.html http://www.nlm.nih.gov/medlineplus/mplusdictionary.html
2.	Co‐text‐defined medical terms	Uptake Replacement	Co‐text
3.	Medical initialisms	TSH RAI	Medical lexicon for abbreviations + co‐text http://www.medilexicon.com
4.	Medication brand names	Cytomel Thyrogen	Drug index resource http://www.rxlist.com/drugs/alpha_a.htm
5.	Colloquial technical terms	Endo Path	Co‐text

Regarding the broadening of the analytical framework, our analysis identified three other categories, namely (i) *medical initialisms* such as *TSH* and *RAI*, identified using a medical lexicon for abbreviations and the surrounding co‐text, (ii) *medication brand names* such as *Cytomel* and *Thyrogen*, determined using an online drug index for brand and generic medication, and (iii) a category that we have named *colloquial technical terminology* consisting of terms such as *endo* (endocrinologist) and *path* (pathology). This latter category was identified using the co‐text. Table 1 provides an overview of these five categories as well as illustrative examples and the methods used for identification.

Using this framework, we chose a second, larger sample from another thyroid forum, consisting of 11 567 words. This second sample was also from the general discussion area and had received 105 replies from eight posters whose experience ranged from 2 posts to 9908 posts. We chose this thread because it had similarities with the first sample in relation to number of posters and their broad range of experience, but also because the topic was different – we wanted to investigate term use when the topic was not related to cancer. The purpose was to see whether the categories worked with this second larger, non‐cancer‐related sample, and whether they were sufficient. The final data set totalled 13 954 words.

## Results

The findings of the coding of the complete data set (Samples 1 and 2) are presented in Table 2. No new categories for terms were found using Sample 2: the analytical framework drawn up using Sample 1 and presented in Table 1 proved sufficient for the complete data set. Our results reveal great diversity within the kinds of terms that the patients are familiar with and use, for example *paratracheal lymph nodes, uptake, RAI, Cytomel* and *frees*.

**Table 2:** Terms used by patients in our data set

	Term type	Examples
1.	Dictionary‐defined medical terms	Ablation, acupuncture, alkaloid, allergy, Alzheimer's, amyloid, anti‐inflammatories, antibodies, antioxidant, arthritis, autoimmune, biopsied, bloodstream, bromelin, caffeine, calcium, calf, central nervous system, cholesterol, cocaine, collar bone, concussion, constipation, cortisone, Crohn's disease, diabetes, dizziness, eczema, endocrine system, extrusions, gland, glaucoma, gums, Hashimotos, hematoma, hyperphosphorylation, hypothyroid, immune system, infarct, inflammation, inflammatory, infusion, injection, insulin, joints, laser therapy, ligament, lozenge, lymph nodes, magnesium, menopause, metabolism, mineral, nausea, nerve, nicotine, orthopedist, osteoarthritis, oxycodone, pancreatic cell, papillary carcinoma thyroid cancer, paratracheal lymph nodes, phosphorylation, pituitary hormone, plantar fasciitis, podiatrist, probiotic, progesterone, prognosis, prolotherapy, prostrate, protein, psoriasis, psoriatic arthritis, quinine, rotator cuff, sciatica, side effects, sinus, spinal stenosis, stage 3 lung cancer, steroids, strychnine, surgeon, tangles, tau protein, tauopathies, tendon, testosterone, thumb, thyroid, thyroiditis, thyroxine, tissue, triglycerides, ulcer, ulcerative colitis, ultra sound, vertebrae
2.	Co‐text‐defined medical terms	Agent [therapeutic agent]
		Collapse [tendon collapse]
		Disc [disc extrusion]
		Drain [put in after surgery]
		Grain [desiccated thyroid medicine]
		Monitoring [monitoring cancer]
		Panel [thyroid panel]
		Procedure [surgery]
		Removal [thyroid removal – thyroidectomy]
		Replacement [replacement medication]
		Replacement [hip replacement]
		Shot [thyrogen shot]
		Shot [cortisone shot]
		Staples [surgical staples]
		Supplement [dietary supplement]
		Suppress [suppress thyroid‐stimulating hormone]
		Tear [ligament tear]
		Test [test results after surgery]
		Trial [medication trial]
		Uptake [radioactive iodine uptake]
3.	Medical initialisms	A/D [antidepressants]
		BP [blood pressure]
		CNS [central nervous system]
		CRP [C‐reactive protein]
		D‐3 [vitamin D3]
		FM [fibromyalgia]
		HC [hydrocortisone]
		HDL [high‐density lipoprotein]
		IT band [iliotibial band]
		LDL [low‐density lipoprotein]
		LDN [low‐dose naltrexone]
		MAOI [monoamine oxidase inhibitors]
		ME [myalgic encephalomyelitis]
		MRI [magnetic resonance imaging]
		OA [osteoarthritis]
		PT [physiotherapist]
		RA [rheumatoid arthritis]
		RAI [radioactive iodine]
		T3 [triiodothyronine]
		T4 [thyroxine]
		TPO [thyroperoxidase]
		TSH [thyroid‐stimulating hormone]
		U/S [ultrasound]
4.	Medication brand names	Advil, Armour, Celebrex, Cytomel, Levoxyl, Lipitor, Pycnogenol, Selenium, Synthroid, Thyrogen, Tylenol, Vicoden
5.	Colloquial technical terms	Alz. [Alzheimer's disease]
		C [vitamin C]
		Chiro [chiropractor]
		Detox [detoxification]
		Doc [doctor]
		Dx'd [diagnosed]
		Endo [endocrinologist]
		Fibro [fibromyalgia]
		Frees [Free T3 and T4]
		Hashi [Hashimotos]
		Hyper [hyperthyroidism]
		Hypo [hypothyroidism]
		Lab and labs [lab test]
		Loz [lozenge, tablet]
		Meds [medicines]
		Ortho docs [orthopedic doctors]
		Palps [palpitations]
		Path [pathology]
		Prolo [prolotherapy]
		Rheumy [rheumatoid arthritis]
		Supp [supplements]
		Syn [Synthroid]
		Tabs [tablets]
		Type 2 [type 2 diabetes]
		Vit E [vitamin E]

## Discussion

The present study was motivated by the potential discrepancy between clinical guidelines which advocate that medical terms should be avoided with patients because of concerns about overestimating what patients can understand, on the one hand, and the ever‐increasing numbers of e‐patients, who use the Internet to equip themselves with relevant condition‐related knowledge, on the other hand. Of course, as pointed out by Elder *et al*.,^33^ use of medical terms may reflect cursory familiarity with terms as opposed to deep understanding, so the implications of the findings should be interpreted cautiously, particularly in relation to what one can conclude about health literacy.

Our findings reveal that patients repeatedly use *dictionary‐defined medical terms* (see Table 2) like *hematoma*,* papillary carcinoma thyroid cancer* and *tauopathies*. Such dictionary‐defined terms are considered by Nation to be the most technical kind of words: ‘Someone who knows these words is likely to have knowledge of that field well beyond knowing the words’.^44^ It would appear that many e‐patients are familiar with these medical terms and are happy to use them actively in public forums.

Many of the terms in all five identified categories belong to the medical specialism of endocrinology, such as *Hashimotos, suppress, TPO, Thyrogen* and *hyper*. In this regard, we would suggest that our empirical results have consequences for current understandings of health literacy. Although health literacy has traditionally been viewed as a quantifiable construct that can be measured through standardized tests of global medical understanding, we suggest that for it to be a more useful construct, it should be considered at the individual level, and be gauged in relation to situated and condition‐specific aspects of a patient's condition. E‐patients’ health literacy could more gainfully be considered in relation to their knowledge of their own specific condition literacy – which in any case is the health literacy that matters to the patient. Thus, for example, the e‐patients in our study may be highly health literate in endocrinology, but not have high health literacy if tested on cardiology‐related terms.

Our empirical findings reveal that e‐patients use a variety of medical terms without glossing, which suggests that other patients’ knowledge of these terms or their ability to cope with them is assumed. Of particular relevance here is our category of *colloquial technical terms*, which recalls the hindclipped terms found in Bowker & Herrera.^17^ This indicates not only familiarity with colloquial medical language but also the assumption of shared knowledge, as these terms, unlike more formal terms such as *carcinoma thyroid cancer*, cannot be found in a dictionary. The example of *endo* [endocrinologist] in our data shows the importance of both co‐text and condition‐specific health literacy, as in other medical contexts it could, for example, mean *endometrium* or *endometriosis*.

Traditionally, medical terms have been defined as features of the discourse of professional experts,^41^ but this study shows that patients with their use of medical terms are using the language of medical experts. With this move into new discursive territory, patients acquire new epistemic roles.^56^ However, term‐savvy patients may meet obstacles as ‘practitioners are not always welcoming of patients who try to “know too much” about medical decisions’.^54^ The shifting power dynamics, reflected in changes in patients’ term use, need further investigation.

We suggest that our article also makes methodological contributions. To undertake the analysis and on the basis of a pilot study, we developed an analytical framework of medical terms. The categories found in our analytical framework underline the importance of a qualitative approach, as a computer would not have identified the categories of co‐text‐defined terms or colloquial technical terms. We suggest that the analytical framework and the means by which it was derived may be transferrable to other empirical studies of functional health literacy, providing a supplementary means of gauging patients’ health literacy.

Regarding the implications of our findings for the clinical situation, one needs to be cautious. First, it is not possible to conclude from our study anything about what the patients understood by the terms they used. Thus, follow‐up studies that move beyond terms and characterize the *knowledge* that e‐patients have would be a very valuable contribution to the current debate on the shifting epistemic status of patients. Furthermore, as this study did not investigate potential differences in terminology use between newcomers and seasoned posters, it would be valuable to investigate posters’ use of medical terminology over time. Moreover, whilst we have shown that the patients in our data set use medical terminology, we are not advocating its indiscriminate use in health communication in general: e‐patients are generally younger, better educated and more affluent than the general population.^57^ Also, as mentioned in our Data section, thyroid patients may be particularly proactive, and this may affect the generalizability of our findings.

However, our findings do suggest that *some* patients with chronic or life‐threatening illnesses have knowledge of and the ability to use complex medical terminology in relation to their specific situation. Health‐care professionals need to be able to assess this and tailor their use of terminology to the individual patient. Like Wittenberg‐Lyles *et al*.^58^ and Dahm,^1^ we suggest an adaptive approach, where patients’ needs and abilities are established and addressed in a patient‐centred way. *Appropriate* pitching of terms can avoid the potentially damaging effects of poor communication brought about by inappropriate (too complex or too simple) use of terms. This is not an easy task. It takes time for a health‐care professional to establish what terminological level is appropriate, which can be problematic in an already time‐pressed consultation.^1^ However, tailoring the terminological level to meet patients’ needs and expectations becomes all the more important as health‐care professionals increasingly use written media such as email to communicate with patients.^59^, ^60^, ^61^ Further research needs to be undertaken to establish how best to meet patients’ terminological needs and expectations, especially as assumptions about patients’ ability to understand terminology are often not precise enough to guide effective message production both orally and in writing.

## Conclusion

Our study reveals the complexity and variety of terms that e‐patients use when communicating online with each other, and suggests a redefinition of health literacy as relating to deep vertical knowledge of a health issue that is relevant to the patient. It also provokes many questions, particularly questions regarding the management of terminological issues in health‐care professional–patient communication. E‐patients who go online and learn the language of biomedicine are not only proactively engaging in their own health care but are also likely to be increasing their functional health literacy. This can lead to a virtuous circle, as greater health literacy in turn supports patient engagement,^62^ which must remain a central goal in health communication.

## Conflict of interests

None.

## Funding

None.
